# Single parenthood and depression: A thorough review of current understanding

**DOI:** 10.1002/hsr2.2235

**Published:** 2024-07-09

**Authors:** Odunayo M. Kareem, Malik O. Oduoye, Priyadarshini Bhattacharjee, Danisha Kumar, Varisha Zuhair, Tirth Dave, Hamza Irfan, Soaham Taraphdar, Saiyad Ali, Onoshioze M. Orbih

**Affiliations:** ^1^ Babcock University Teaching Hospital Ilishan‐Remo Nigeria; ^2^ Department of Research The Medical Research Circle (MedReC) Goma Democratic Republic of the Congo; ^3^ Cambridge University Hospitals NHS Foundation Trust Cambridge UK; ^4^ Dow University of Health Sciences Karachi Pakistan; ^5^ Department of Medicine Jinnah Sindh Medical University Karachi Pakistan; ^6^ Bukovinian State Medical University Chernivtsi Ukraine; ^7^ Department of Medicine Shaikh Khalifa Bin Zayed Al Nahyan Medical and Dental College Lahore Pakistan; ^8^ College of Medicine and Sagore Dutta Hospital Kolkata India; ^9^ Saidu Medical College Saidu Sharif Pakistan; ^10^ Afe Babalola University Ado‐ekiti Ado‐Ekiti Ekiti State Nigeria

**Keywords:** consequences, depression, measures, modalities, prevalence, single parents

## Abstract

**Background:**

Single parenthood is becoming increasingly common in today's society for various reasons such as divorce, the death of a spouse, or the choice of parenthood. Regrettably, there seems to be no significant concern among world leaders regarding depression arising from single parenting.

**Aim:**

This article aimed to explore the prevalence of depression in single parents, the factors contributing to it, and its effects on their physical and emotional well‐being. Additionally, it aims to investigate the long‐lasting effects of depression in single parents, effective therapeutic approaches to tackle these issues and offer proactive suggestions for relevant global stakeholders.

**Methodology:**

A selection of studies was identified through electronic databases such as PubMed, Embase, and PsycINFO databases. The search strategy encompassed terms related to single parenthood, depression, mental health, prevalence, risk factors, and treatment modalities. Included studies comprised of peer‐reviewed research articles, systematic reviews, meta‐analyses, and observational studies published in English.

**Result:**

Today, there is a growing prevalence of single parenthood due to a range of factors, including divorce, the loss of a partner, and intentional decisions regarding single parenthood. However, this transition comes with challenges, including the risk of developing depression. Depression is a serious mental health condition affecting many individuals worldwide. Raising a child alone increases the likelihood of developing depression for the parent due to the increased burden and responsibilities. Such parents tend to have low self‐esteem, suicide/suicide attempts, and so forth and children born by those parents are vulnerable to depression, physical abuse, infections, etc.

**Conclusion:**

Future research should focus on identifying effective interventions for treating depression among single parents and improving the availability of mental health facilities for this vulnerable population, especially in places with a high prevalence of depression. Mental health physicians in collaboration with obstetricians and gynecologists across the globe should offer counseling and mediation services during pre‐conception care visits for both single and partnered parents.

## 1 INTRODUCTION

Single parenthood is becoming increasingly common in today's society for various reasons such as divorce, the death of a spouse, or the choice of parenthood.^1^, ^2^, ^3^, ^4^ However, this transition comes with its own set of challenges, including the risk of developing depression.^5^, ^6^ Depression is a significant mental health disorder that affects a considerable portion of the global population, particularly in regions such as the UK, the United States, Canada, Germany, Asia, and South Africa.^1^, ^2^, ^3^, ^4^, ^5^, ^6^, ^7^, ^8^, ^9^, ^10^, ^11^, ^12^, ^13^, ^14^, ^15^ Single parents are more likely to experience depression due to the stress and responsibilities of raising a child alone.^4^, ^9^ Some of the consequences of depression due to single parenthood include; low self‐esteem, isolation, and food deprivation that can lead to chronic weight loss, reduced sociocultural activities and religious practices as well as suicide attempts and suicide, and so forth.^6^, ^16^ Furthermore, children of parents who are suffering from depression also experience various psychological challenges such as depression, antisocial behaviors, and personality disorders. They are also susceptible to issues like malnutrition, child abuse, trafficking, and sexual exploitation. Additionally, they may face problems such as low school performance, school dropouts, and infections like malaria, tuberculosis, measles, cholera, typhoid, pneumonia, and physical disabilities. In the most severe cases, these challenges can lead to eventual death.^17^, ^18^, ^19^, ^20^

Regrettably, there seems to be no significant concern among world leaders regarding depression arising from single parenting.^21^, ^22^ Without appropriate attention, the global prevalence of depression arising due to single parenting could lead to increased rates of mortality and morbidity. Consequently, addressing this critical issue necessitates the composition of the present review. This present review is therefore aimed to explore the prevalence of depression in single parents, the factors contributing to it, and its effects on their physical and emotional well‐being. Additionally, it aims to investigate the long‐lasting effects of depression in single parents, effective therapeutic approaches to tackle these issues and offer proactive suggestions for relevant global stakeholders.

## 2 METHODOLOGY

This literature review aimed to examine the prevalence of depression among single parents and its implications on mental health, drawing from a selection of studies identified through PubMed, Embase, and PsycINFO databases. The search strategy encompassed terms related to single parenthood, depression, mental health, prevalence, risk factors, and treatment modalities. Included studies comprised peer‐reviewed research articles, systematic reviews, meta‐analyses, and observational studies published in English.

Our review focused on studies that specifically addressed single parents, encompassing both single mothers and single fathers, across various demographics and geographical locations. The inclusion criteria for this review encompassed peer‐reviewed research articles, systematic reviews, meta‐analyses, cohort studies, and observational studies. These studies were required to investigate single parents, comprising both single mothers and single fathers, across all age groups and geographical locations. The primary focus was on exploring the prevalence, risk factors, consequences, and treatment modalities of depression within this population. Articles had to be published in English to be considered eligible for inclusion. Conversely, nonpeer‐reviewed literature such as gray literature, conference abstracts, and editorials were excluded from consideration. Additionally, studies that did not directly address depression among single parents or those focusing solely on partnered parents were excluded to maintain relevance to the research question. The criteria were designed to ensure that the selected literature provided comprehensive insights into the intersection of single parenthood and depression, facilitating a thorough understanding of this important topic.

Studies explored the multifaceted nature of depression in single parents, highlighting its elevated prevalence compared to partnered parents. Single mothers, in particular, were found to be at a heightened risk, facing challenges such as economic deprivation, social isolation, and inadequate social support. Financial difficulties emerged as a prominent contributing factor to depression among both single mothers and fathers, impacting their mental well‐being and parenting capabilities. The review also delved into the long‐term consequences of depression among single parents, emphasizing its enduring effects on their emotional and physical well‐being. Chronic depression was identified as a significant concern, often stemming from prior marital difficulties, economic hardships, and societal stigma. The review underscored the need for effective treatment modalities to address depression in single parents, recognizing the potential positive outcomes for both parents and their children. Furthermore, the review highlighted the unique challenges faced by single fathers, including lower average incomes, higher rates of unemployment, and societal assumptions about their role as primary caregivers. Despite these challenges, single fathers were found to be less likely to seek formal assistance, often relying on informal support networks for help with parenting, finances, and health. Overall, this literature review provides insights into the complex interplay between single parenthood and depression, shedding light on the need for targeted interventions and support services to alleviate the mental health burden experienced by single parents and promote their overall well‐being.

## 3 REVIEW OF THE LITERATURE

### 3.1 Prevalence of depression among single parents

Single‐parent households house accommodate around 14% (320 million) of the world's 2.3 billion children. According to the statistics, 24% of children in the UK, 27% of children in the United States, 20% in Canada, 18% in France, and 43% of South African children live in single‐parent households. These households are primarily led by single mothers; however, single‐father households also form a significant proportion, with about 300,000 households in the UK, 330,000 in Canada, and 2.6 million families in the United States.^1^, ^2^ In Asia, notably in South Korea, 9.3% of households are managed by single parents, with single fathers heading 21.1% of these households.^3^ Similarly in Japan, single‐parent households make up 8.7%, with single mothers heading 90% of them, and single fathers managing the remaining 10%.^4^ The number of single‐parent households, led by both single mothers and fathers, is increasing rapidly. In the USA, in a 50‐year period from 1960 to 2010, single‐mother households have increased by fourfold, and single‐father households have increased by ninefold.^7^ However, research on single‐parent households predominantly centers around single mothers. Single fathers are crucially neglected in the aspect of research on their health and well‐being.^1^, ^2^ Table 1 presents summary of previous studies investigating the relationship between single parenthood and depression.

**Table 1:** Summary of previous studies investigating the relationship between single parenthood and depression.

	Study title	Study type	Objective	Study population	Country	Results
1.	Mental health among single and partnered parents in South Korea (2017)	Cross‐sectional survey	To examine mental health differences between single and partnered parents, considering social factors and gender variations.	141 single fathers, 5014 partnered fathers, 407 single mothers, and 6462 partnered mothers	South Korea	Single parents showed significantly poorer mental health compared to partnered parents, with higher odds of depressive symptoms (OR: 2.02, 95% CI: 1.56–2.63), suicidal ideation (OR: 1.69, 95% CI: 1.27–2.25), and any of the three mental health conditions (OR: 1.74, 95% CI: 1.38–2.20).
2.	Mental health in young mothers, single mothers and their children (2019)	Cohort study	To scientifically examine how early motherhood and single motherhood influence maternal mental health and the well‐being and developmental outcomes of children.	1723 Mothers	Sweden	Single mothers at childbirth didn't demonstrate elevated risk for postpartum depression symptoms (OR 1.000, CI 0.495–2.020). Additionally, they didn't report higher levels of externalizing or internalizing problems in their children compared to cohabitating mothers (OR 1.422, CI 0.636–3.183; OR 1.985, CI 0.934‐4.217).
3.	Prevalence of Psychiatric Disorder in Lone Fathers and Mothers: Examining the Intersection of Gender and Family Structure on Mental Health (2011)	Cross‐sectional Survey	To investigate the prevalence of psychiatric disorders in lone fathers and mothers, with a specific focus on understanding how the intersection of gender and family structure influences mental health outcomes, particularly emphasizing the mental health of lone fathers.	769 Single father and 1964 Single mothers, 5340 Cohabitating Father, and 5505 Cohabitating Mothers	Canada	Single fathers and single mothers show higher prevalence rates of mood disorders and substance use disorders (SUDs) compared to their married counterparts. Among single parents, mothers have a higher prevalence of anxiety disorders (10.7% vs. 4.9%) and mood or anxiety disorders (19.9% vs. 11.1%) than fathers.
4.	Impact of economic problems on depression in single mothers: A comparative study with married women (2018)	Cross‐sectional Survey	The objective of this study was to examine the prevalence of depression among single mothers and identify the risk factors that contribute to its occurrence.	195 single mothers, 357 married mothers	South Korea	The prevalence of depression showed a notable difference between single mothers (33%) and the control group (8%). In single mothers, factors such as young age, low income, residential instability, high stress, and elevated alcohol‐related problems were found to be associated with depression.
5.	Psychosocial factors associated with symptoms of depression, anxiety and stress among single mothers with young children: A population‐based study (2019)	Cross‐sectional Survey	The objective of this study was to investigate the psychosocial factors associated with symptoms of depression, anxiety, and stress among single mothers with young children.	517 Single mothers, 6408 partnered mothers	Germany	Single mothers (*n* = 517) reported significantly higher rates of depressive or anxiety symptoms (30%) and general stress (37%) compared to partnered mothers (*n* = 6408; *p* < 0.0001). Further analysis indicates that single mothers are twice as likely to experience symptoms of depression or anxiety (OR 1.9, CI 95% 1.4–2.5). Inadequate social support and a history of partner or childhood maltreatment emerged as consistent risk factors across all outcomes.
6.	Depression and quality of life for women in single‐parent and nuclear families (2009)	Cross‐sectional Survey	This study aimed to achieve two primary objectives. Firstly, it sought to determine the predictors influencing perceived quality of life. Secondly, it aimed to analyze and compare the differences in quality of life, depression levels, and family income between women hailing from single‐parent families and those from two‐parent families.	107 women from bi‐parent families, 33 women from single‐parent families	Mexico	The results reveal that women from single‐parent families demonstrate a lower quality of life (*Z* = −2.224, *p* = 0.026), reduced income (*Z* = −2.727, *p* = 0.006), and elevated depression levels (*Z* = −6.143, *p* = 0.001) compared to women from bi‐parental families.
7.		Cross‐sectional Survey	The objective of this study was to compare the prevalence rates of depression, as well as other mood and anxiety disorders, between single and married mothers in Singapore.	10 Single or unmarried women, 1510 married women, 97 divorced or separated women and 21 widowed women	Singapore	Single mothers exhibited markedly higher odds of experiencing mood disorders (OR = 5.28) compared to married mothers.
8.	Stress, social support and depression in single and married mothers (2003)	Secondary analysis of survey	The objective of this study was to investigate the influence of stress and social support on the association between single‐parent status and depression.	725 single mothers and 2231 married mothers	Canada	Compared to married mothers, single mothers showed higher rates of recent depression episodes, increased chronic stress levels, and more frequent recent life events and childhood adversities. Stress and social support accounted for almost 40% of the association between single‐parent status and depression. Additionally, stress had a differential impact on depression based on family structure, with married mothers exhibiting a stronger correlation between life events and depression.
9.	Depressive and anxiety disorders among single mothers in Dhaka (2020)	Cross‐sectional survey	To investigate the prevalence and proportions of depressive and anxiety disorders among single mothers residing in Dhaka, Bangladesh.	156 single mothers	Bangladesh	In Dhaka, 48.8% of single mothers experienced either depressive and/or anxiety disorders, with 17.3% diagnosed with depressive disorder, 21.2% with anxiety disorders, and 10.3% with both.

Abbreviations: CI, confidence interval; OR, odds ratio.

A significant degree of depression, anxiety, and stress have been linked to single motherhood.^8^ Studies indicate that the rate of depression experienced by single mothers within a 12‐month period is nearly twice as high as that of married mothers.^5^, ^6^, ^9^, ^10^, ^11^ Furthermore, single mothers exhibit a threefold higher incidence of depressive phases compared to other demographic groups, with a 12‐month prevalence rate of depressive episodes at 7%.^9^ Moreover, the symptoms of depression or anxiety are twice as high in mothers who are single compared to partnered mothers.^8^ While the manuscript does not provide a specific prevalence rate of depression among single parents, the incidence of depressive symptoms that are significant clinically is higher in countries such as Cyprus, where 38.9% of single mothers reported clinical symptoms of depression.^6^ According to Eurostat, the prevalence of depression in the overall population during a lifetime is almost three times lower than the prevalence observed among single mothers.^6^ Additionally, mothers of young children who are single experience symptoms of depression, anxiety, and stress.^8^ Research findings indicate that a significant portion of the association between single parenthood and depression can be attributed to variations in stress levels and access to social support.^10^ Furthermore, single mothers who are economically inactive, receive a single mother's allowance, or have a lower monthly family income are at greater risk of experiencing depression.^6^ Likewise, single mothers with a junior high school education exhibit a higher prevalence of clinically significant depressive symptoms than those with a university education.^6^ As for single fathers, studies show that they are twice likely to have poorer mental and self‐rated health than single mothers, but are half likely to get access to healthcare.^2^ Research shows that single fathers have much less social capital, that is, social relations and connections that help their mental and physical health, well‐being, and productivity.^11^ Single fathers also have a much higher rate of smoking and binge drinking as compared to partnered fathers. All these factors contribute to an increased rate of depression, along with morbidity and mortality factors in single fathers in comparison with partnered fathers.^2^

### 3.2 Single parenthood and its implications on mental health

Single parenthood can profoundly impact the mental well‐being of individuals. Transitioning into single parenthood can create significant distress for parents and negatively affect the overall well‐being of their children. Consequently, this can contribute to perpetuating inequality across generations.^6^ While divorce and separation are typical routes to single parenthood, the experience of losing a spouse was observed to occur more frequently among single fathers than single mothers, which further contributes to their psychological stress.^2^ Frequently, single parents find themselves in the dual position of being the primary caregiver for their family while also shouldering the responsibility of being the primary provider,^6^, ^12^ leading to an increase in economic, social, and psychological stress. This domino effect can result in lesser finances and communal assistance, leading to parenting adjustment and behavioral issues.^6^ In many societies, single families are viewed as abnormal, defective, and incomplete families. This not only adds to the emotional distress of the parents but also pushes the families into hiding their single‐family status and exposing themselves to stressful situations.^15^

The perception of inadequate social support adds to the decline in mental well‐being among single mothers, who show a greater tendency to encounter mental health issues in comparison to mothers in partnered relationships.^23^ Future research should investigate the dynamics of financial hardships among various family structures, examine its impact on mental health over time, and identify factors that may mitigate the connection between social disadvantage and the presence of mental health problems.^15^ Financial hardship is a prominent factor linked to diminished mental health among single mothers.^15^ Single parenthood negatively affects the mental health of individuals, and the link between single parenthood and mental well‐being may be mediated by sociodemographic, financial, and social support variables.^15^ Studies have revealed high levels of psychological problems among single mothers in comparison to mothers in partnerships due to psychosocial and socioeconomic risk factors such as stress and specific personality characteristics.^6^, ^9^, ^10^, ^11^, ^12^, ^13^ Single mothers are vulnerable populations and are more likely to develop poor mental and physical health.^23^ Ensuring their psychosocial well‐being necessitates the provision of essential social support. Mothers who perceive inadequacy of support or express discontent with the quality of assistance they receive are vulnerable to experiencing psychological distress. Single motherhood, in particular, is associated with elevated stress levels.^6^ Several studies investigating the relationship between social support and single motherhood have highlighted that single mothers often rely on their family and close acquaintances for emotional assistance and practical help.^6^ Medical interventions focused on enhancing self‐efficacy among single mothers dealing with depression can contribute to favorable health outcomes and facilitate positive behavioral changes.^23^ Gaining insights into the underlying physiology of psychological problems among single mothers holds significance in shaping policy development. Implementing strategies for effective financial management has the potential to reduce the likelihood of mental instability among both single mothers and their children. Notably, financial hardship emerges as a pivotal factor influencing the well‐being of single mothers.^6^ Additionally, the greater utilization of services among single mothers can be attributed to a higher prevalence of psychiatric disorders.^24^ The primary factor that differentiates the utilization of service by married and single mothers is the level of need, with single mothers experiencing higher rates of psychopathology.^24^ As a result, single‐parent mothers exhibit a two to three times higher likelihood of utilizing services.

Despite having higher incomes than single mothers, single fathers may encounter difficulties in handling the dual responsibilities of being the primary provider and caregiver due to their less common involvement in solo parenting roles.

^2^ Furthermore, research has demonstrated that marriage exerts a safeguarding influence on the mental well‐being and social conduct of men.^2^, ^4^, ^16^, ^25^ Fathers in partnered relationships display a decreased likelihood of participating in risky behaviors compared to those who are single.^2^ Moreover, the gendered role of the sole provider along with male pride makes pushes many single fathers to seek help in parenting, finance, education, and health from informal social networks like male‐oriented support groups or extended families rather than official governmental systems.^12^

### 3.3 Factors contributing to depression among single parents

Single parents, especially mothers, are at greater risk of developing depression than the average population.^16^ This is because of the added pressures faced by single parents, including challenges like financial difficulties and social isolation.^16^ Economic deprivation contributes significantly to depression among single mothers.^21^ Due to their single‐parent status, single mothers are likely to face financial difficulties, such as a lack of resources.^21^ Low monthly income and social discrimination are the noteworthy factors affecting depression in single mothers.^21^ While single mothers encounter economic inconveniences more, it is essential to acknowledge that single fathers also encounter challenges raising children and may face social biases.^21^ Studies found that self‐esteem issues were a significant factor contributing to depression in single fathers.^21^ In addition, adolescents whom single mothers raise encounter a higher number of childhood stressors and face an elevated risk of developing depression.^26^ The precise mechanisms elucidating the connection between single‐mother households and depression are not fully understood. However, it has been observed that higher levels of rumination facilitate the association of single motherhood with increased symptoms of depression in young individuals.^26^

Single fathers, too, are shown to have more economic stressors than partnered ones, as they have a lower average income and a higher rate of unemployment.^2^, ^4^ Moreover, social stigma is higher in the case of single fathers than single mothers, with societal assumptions about the role of the primary caregiver. Due to this, single fathers are reported to have faced more bureaucratic hurdles than single mothers, which adds to their stress levels.^2^ Single fathers with young children often suffer from lack of sleep which aids in psychological stress, leading to depression.^4^ Studies in Germany show that single fathers not only have more medically diagnosed depression cases than partnered fathers but their psychological burden and prevalence of mental health disorders are higher compared to single mothers.^27^

### 3.4 The impact of depression on the emotional and physical well‐being of solo parents

Depression is a complicated mental health issue for single parents to overcome.^5^, ^6^, ^10^, ^11^, ^12^, ^13^, ^14^, ^15^, ^23^ There is a greater likelihood of a single mother experiencing an episode of depression compared to married ones.^10^ Moreover, the stress of single parenting increases the likelihood of developing depression.^16^ Depression can lead to decreased quality of life for single parents^16^ and can harm their physical and emotional well‐being.^15^, ^16^ The situation is further complicated by the fact that single mothers tend to have lesser perceived social aid, limited societal participation, and reduced connection with family and friends. Additionally, they are often victims of chronic stress, disturbing events in life, and childhood traumas.^10^ Single mothers of young children are especially susceptible to mental health disorders due to financial challenges, social pressures, or distal hardships.^8^ According to studies, single mothers display the highest prevalence of conspicuous depressive symptoms and the consideration of suicidal thoughts.^3^, ^22^ Self‐efficacy theory holds promise for improving self‐efficacy in single mothers with depression, which can encourage positive health habits and behavior changes.^23^ Unmarried fathers are more prone to taking part in risky behaviors, including engaging in sexual activities, driving recklessly, having an unhealthy diet, smoking, drinking excessively, using substances, and delaying seeking medical help.^2^ Studies have also shown single fathers have the lowest rate of utilizing health services for their children, compared to single mothers or partnered parents.^12^ To mitigate the existing disparities between single and partnered parents related to mental well‐being, it is crucial to implement suitable social support programs and establish effective screening measures.^8^

### 3.5 Long‐term consequences of depression among single parents

The risk of developing depression among single parents is much greater than among married or partnered parents.^28^ Factors such as discrimination, monthly income, and economic hardships are related to the onset of depression among single parents.^21^ Single mothers may have low self‐esteem and a lack of social support, which increases their risk of experiencing depression onset.^21^, ^28^ Depression in single fathers can be attributed to both financial struggles and the challenges of parenting.^21^ Studies have also shown that financial hardship increases the likelihood of chronic depression.^28^ Single parents have a higher risk of financial instability than partnered parents.^2^ Furthermore, chronic depression among single parents often originates from prior marital difficulties or widowhood.^28^ Additionally, single parents have a higher probability of experiencing humiliating or entrapping severe life events,^28^ which can contribute to depression. Although chronic depression decreases as the duration of single parenthood increases, single parents still face a higher risk.^28^ The long‐term consequences of depression in single parents are a significant concern and have considerable implications for psychiatric and social policies.^9^

### 3.6 Effective treatment modalities for addressing depression in single parents

Depression can hinder the ability to effectively engage in successful single parenting and can have enduring consequences on a child's physical, social, and emotional well‐being.^29^ Single mothers exhibit a higher likelihood of experiencing depression and may necessitate more intensive treatment approaches.^29^ Unfortunately, many single mothers drop out of treatment before remission.^29^ Research findings have demonstrated that when mothers experiencing depression achieve complete remission after undergoing 3 months of treatment, there is a notable enhancement in their children's disorders.^29^ These findings indicate a potential link between having a father in the household, the remission of maternal depression, and positive outcomes for the child.^29^ However, only onethird of the mothers who underwent remission over the 3 months indicated the need for more effective treatment options for depression in single parents.^29^ Thus, considering the factors affecting maternal remission rates is important while predicting child outcomes.^29^ Despite this, the effectiveness of treatment for depression in single parents is not mentioned in the text, yet it is possible to treat depression in single parents effectively.^23^

## 4 CONCLUSION AND RECOMMENDATIONS

Depression within the context of single parenting is a significant and concerning mental health issue that can harm individuals and their families. This review highlights the prevalence of depression, stress, and anxiety among single parents than partnered parents. Financial instability, inadequate social support, and lower educational levels are among the sociodemographic factors linked to depression in single parents. The number of studies on single fathers is significantly lesser compared to studies on single mothers. The review also revealed that treatment for depression in single parents is not well‐documented. In addition, the review notes that many single mothers drop out of treatment before reaching remission, which is concerning. The findings of this study suggest that single parenthood negatively affects mental health and that the link between single parenthood and mental health problems may be intermediated by sociodemographic, financial, and social variables. This study also highlights the importance of providing effective treatment and support to single parents to improve their mental health and overall well‐being.

Future research should focus on identifying effective interventions for treating depression among single parents and improving the availability of mental health facilities for this vulnerable population, especially in places with a high prevalence of depression.^14^ The need for qualitative research exploring various facets of depressed single parents is warranted in the world, especially in places with a high prevalence of depression. Documentation of such interview‐based research is imperative to provide future guidelines to mitigate the problem mentioned above as well as orient physicians especially mental health physicians, researchers, and patients towards crisis management. The policy‐makers in the world, especially in countries with a high prevalence of depression should implement a sustainable family policies such as transfer payments, time and infrastructure policies in promoting the well‐being of single parents.^22^, ^30^ Researchers have determined that the introduction of a sustainable family policy can effectively counter the challenges associated with living alone, ultimately mitigating the influence of poverty and reducing the occurrence of psychological issues.^30^ All Government agencies (at the national and subnational levels) and international organizations like the WHO, UNESCO, UNFPA, UNICEF, and so forth should provide subsidized childcare, tax relief for single parents worldwide as well as an allowance for both parents and their children.^31^

Rattay et al.^22^ suggested that; in addition to the provision of job opportunities and empowerment for single parents, government should also provide flexibilization of working time models, that is; reducing working hours, temporary work leaves, designed to accommodate the varying time demands experienced during different life phases.^32^, ^33^ Furthermore, world leaders especially those in the UK, United States, Germany, and Asian region should implement the “Frühe Hilfen Netzwerke”^34^ strategy in their countries through mental health physicians, psychologists as well as the social health workers in their countries and regions. “Frühe Hilfen” strategy is a German word that simply means "Early‐Help”.^34^ It is an Austrian model developed since the year 2015 by the Austrian National Public Health Institute (GȪG) on the basis of global findings and systematic evaluations of practical experiences.^34^ “Frühe Hilfen” is a regional networking service aimed at early interventions for families in need. The “Frühe Hilfen” initiative employs a collaborative approach involving various professionals and sectors. It places special emphasis on forming networks, particularly with services within the health and social sectors.^32^ Therefore adopting the “Frühe Hilfen” model for single parents would them during stressful conditions such as depression and other psychological problems. Mental health physicians across the globe should step up in their mental health advocacy about depression among young people especially those getting ready for marriage and marital responsibilities. In addition, mental health physicians in collaboration with obstetricians and gynecologists across the globe should offer counseling and mediation services during pre‐conception care visits for both single and partnered parents. This would go a long way in preventing depression among parents as well as improving their self‐esteem positively and preventing them from stigmatization in their communities.

## AUTHOR CONTRIBUTIONS

**Odunayo Mayowa Kareem**: Conceptualization; resources; writing—original draft; writing—review & editing. **Malik Olatunde Oduoye**: Conceptualization; project administration; writing—original draft; writing—review & editing. **Priyadarshini Bhattacharjee**: Writing—original draft; writing—review & editing. **Danisha Kumar**: Writing—original draft; writing—review & editing. **Varisha Zuhair**: Writing—original draft; writing—review & editing. **Tirth Dave**: Project administration; writing—original draft; writing—review & editing. **Hamza Irfan**: Writing—original draft; writing—review & editing. **Soaham Taraphdar**: Writing—original draft; writing—review & editing. **Saiyad Ali**: Writing—original draft; writing—review & editing. **Onoshioze Martha Orbih**: Writing—original draft; writing—review & editing.

## CONFLICT OF INTEREST STATEMENT

The authors declare no conflict of interest.

## TRANSPARENCY STATEMENT

The lead author Tirth Dave affirms that this manuscript is an honest, accurate, and transparent account of the study being reported; that no important aspects of the study have been omitted; and that any discrepancies from the study as planned (and, if relevant, registered) have been explained.

## Data Availability

The data that support the findings of this study are available from the corresponding author upon reasonable request.
