# Correction to “Single Parenthood and Depression: A Thorough Review of Current Understanding”

**DOI:** 10.1002/hsr2.73280

**Published:** 2026-09-25

O. M. Kareem, M. O. Oduoye, P. Bhattacharjee, D. Kumar, V. Zuhair, T. Dave, H. Irfan, S. Taraphdar, S. Ali, O. M. Orbih, “Single Parenthood and Depression: A Thorough Review of Current Understanding,” *Health Science Reports* 7, no. 7 (2024): e2235, https://doi.org/10.1002/hsr2.2235

Corrections

Some errors were identified related to referencing, citation accuracy, and content support. The following corrections have been made:

1. Removal of self‐citation and associated references
  References 17–20 have been removed due to inappropriate self‐citation and lack of relevance to the corresponding text. The associated statements have also been revised accordingly.
2. Addition of references in the Introduction
  The following references have been added to strengthen statements regarding maternal depression and mental health outcomes:
  o Talati A et al., 2007
  o Agnafors S et al., 2019
  o Subramaniam M et al., 2014
  o Nahar JS et al., 2020
3. Addition of references in Section 3.1 (Prevalence of depression among single parents)
  The following reference has been added:
  o Mental Health in the EU—Key facts, figures and activities (European Commission)
  o Additionally, the following study has been included:
  o Ringbäck Weitoft G et al., 2004
4. Addition of references in Section 3.3 (Factors contributing to depression among single parents)
  The following references have been added to strengthen statements on economic and psychosocial determinants:
  o Kim GE et al., 2018
  o Mental health of single mothers in Australia (Journal of Public Mental Health)
  o Dhungel B et al., 2023
  o Daryanani I et al., 2017
5. Correction and addition of references in Section 3.3
  The following reference has been corrected:
  o Rattay P et al., 2017
  o Additional supporting references have been included:
  o Müters S et al., 2013
  o Von der Lippe E et al. (German population‐based study)
6. Correction in Section 3.6 (Treatment modalities)
  The reference supporting remission of maternal depression has been corrected to:
  o Talati A et al., 2007
7. Table 1 corrections
  o Missing study titles have been added where previously omitted
  o Corresponding citations have been inserted for all listed studies

These corrections do not affect the conclusions of the article.

We apologize for these errors.

